# The Impact of Transient Hepatic Attenuation Differences in the Diagnosis of Pseudoaneurysm and Arteriovenous Fistula on Follow-Up CT Scans after Blunt Liver Trauma

**DOI:** 10.3390/diagnostics4030129

**Published:** 2014-09-10

**Authors:** Andreas Hjelm Brandt, Caroline Ewertsen, Kristoffer Lindskov Hansen

**Affiliations:** Department of Radiology, Copenhagen University Hospital Rigshospitalet, Blegdamsvej 9, 2100 København Ø, Denmark; E-Mails: caroline.ewertsen@dadlnet.dk (C.E.); lindskov@gmail.com (K.L.H.)

**Keywords:** abdomen, CT, liver, trauma, vascular injury

## Abstract

A feared complication to liver trauma is delayed vascular complication, such as pseudoaneurysm and arteriovenous fistula (PS/AF) seen as focal enhancement on contrast-enhanced computed tomography (CT) in the arterial phase. A hyperdense area termed transient hepatic attenuation difference (THAD) representing altered hepatic blood flow can be seen in the arterial phase near the liver lesion. The objective of this study was to describe THAD and PS/AF on follow-up CT after blunt liver trauma, and to evaluate if THAD influenced the evaluation of PS/AF. Three radiology residents retrospectively evaluated scans of 78 patients. The gold standard for PS/AF was an evaluation by an experienced senior radiologist, while THAD was a consensus between the residents. PS/AF was present in 14% and THAD in 54%. THAD was located in the periphery of the lesion with hazy borders and mean HU levels of 100, while PS/AF was located within the lesion with focal enhancement and mean HU levels of 170 (*p <* 0.05). In evaluation of PS/AF, the likelihood of agreement between the observers and the gold standard was 89% when THAD was present, and 98% when THAD was absent (*p =* 0.04). THAD is common and can hamper the evaluation of PS/AF.

## 1. Introduction

The liver is the most commonly injured organ after blunt abdominal trauma, with a reported prevalence from 4.2% to 8.0% [1,2]. Within the last decade, treatment of liver trauma has changed towards a more conservative approach [3], and computed tomography (CT) is today the diagnostic method of choice for primary evaluation of liver trauma in hemodynamically stable patients [4].

In several centers, a follow-up CT scan on patients with CT confirmed liver injuries is performed two to seven days after hospital admission to ensure that no complicating injuries are present. Common complications to non-surgical management are hemorrhage, biloma and hepatic abscess. Hemorrhage is primarily caused by a delayed vascular complication (DVC), such as pseudoaneurysm or arteriovenous fistula (PS/AF) and subcapsular hematoma, occurring in up to 5.9% of the patients [5,6,7]. Even though the exact mortality rate of untreated DVC is unknown, several fatal outcomes have been reported [8,9,10,11,12].

With the improved temporal resolution, faster data acquisition as well as larger detector coverage of CT scanners, it is a normal procedure to evaluate the liver in more than one phase. Patients with blunt hepatic traumas are often evaluated with contrast-enhanced CT in both the arterial and the venous phase, where PS/AF is primarily seen in the arterial phase. When a PS/AF is suspected, digital subtraction angiography (DSA) may be performed. DSA has a higher sensitivity and specificity than CT for these injuries [13]. Furthermore, DSA allows a more precise identification and selective embolization of the appropriate branch vessel as opposed to surgical ligation of a main hepatic artery or hepatic resection [14].

Hyperdense areas in the liver parenchyma in relation to liver lesions are also commonly seen in the arterial phase of the follow-up CT scan. This phenomenon, termed transient hepatic attenuation difference (THAD), represents an altered blood flow, where the arterial flow increases due to a parenchymal inflammation and/or a diminished portal inflow [15,16,17]. THAD has been associated with liver trauma, malignant and benign focal lesions, cholecystitis, cholangitis and Budd-Chiari [17].

The aim of this study was to describe and evaluate THAD and PS/AF found on follow-up CT scan after blunt liver trauma, and to evaluate if THAD influenced the interpretation of PS/AF on follow-up CT scans.

## 2. Material and Methods

### 2.1. Patients

A retrospective search was performed for patients with confirmed blunt liver trauma admitted to the Department of Surgical Gastroenterology at Copenhagen University Hospital, Rigshospitalet between November 2008 and November 2012. The patients were identified by the International Classification of Disease code (Laesio traumatica hepatis et vesicae felleae/DS361) in the discharge letter. A subsequent review was performed in the web-based archive control system RIS/PACS at the Department of Radiology at Copenhagen University Hospital, Rigshospitalet to select all patients with an initial CT scan and a follow-up CT scan. Of the 226 patients admitted with traumatic liver lesions during the observation period, 94 patients had suffered blunt liver trauma, and of these, 78 patients (43 males and 35 female, mean age: 34.7 years, range: 5–85 years) met the inclusion criterion with an initial and a follow-up CT scan. Among the included patients were 14 pediatric patients (mean age: 10.1 years, range: 5–17 years).

### 2.2. Imaging

The initial CT scan was performed with scan protocol depending on local guidelines at the hospital, where the patient was examined initially. Forty-one patients had their initial CT performed at Copenhagen University Hospital, Rigshospitalet using a specific trauma protocol on a 64-slice helical CT scanner. Contrast agent Omnipaque (GE Healthcare, Oslo, Norway) in a dose of 100 mL (350 mg iodine/mL) was injected intravenously with a flow of 3 mL/s. The delay time was 55 s and reconstructions were done in axial, sagittal and coronal planes with slice thickness of 3 mm.

All follow-up CT scans were performed at Copenhagen University Hospital, Rigshospitalet on a 64-slice helical CT scanner. Contrast agent Omnipaque (GE Healthcare, Oslo, Norway) in a dose of 130 mL (350 mg iodine/mL) was injected intravenously with a flow of 4 mL/s. For the pediatric patients, the contrast agent Omnipaque (GE Healthcare, Oslo, Norway) was injected in a dose corresponding to 1.5 mL/kg (350 mg iodine/mL) with a maximal flow of 2 mL/s. The follow-up CT examination included an abdominal series without contrast enhancement, followed by a contrast enhanced series in two phases: arterial and venous. Automatic tracing was done with the region of interest placed in the abdominal aorta and the threshold set to a value of 150 Hounsfield units (HU). The arterial phase was reached with a delay of 15 s and the venous phase with a delay of 85 s, after reached threshold value. Reconstructions were done in axial, sagittal and coronal planes with slice thickness of 3 mm. Between the initial CT scan and the follow-up CT scan was a median of 5 days (mean: 4.6 days, range: 1–9 days).

### 2.3. Evaluation

The expected difference between correct evaluations of the follow-up CT scans for patients with and without THAD was assumed to be 10%. Applying this in a sample size calculation, it was shown that 62 evaluations of follow-up CT scans of patients with and without THAD were sufficient to reject the null hypothesis. 50% of the included patients were expected to present THAD, thus, three observers were sufficient when 78 patients were included in to the study.

Three residents in radiology retrospectively evaluated the follow-up CT scans of 78 patients with blunt liver trauma for the presence of PS/AF and THAD. The evaluation was done with the evaluators blinded to the results of the follow-up scan and to the knowledge of other exams performed afterwards, e.g., DSA. Inspection of the initial CT to locate the lesion was allowed and all phases of the follow-up CT were used for the evaluation.

**Figure 1:**
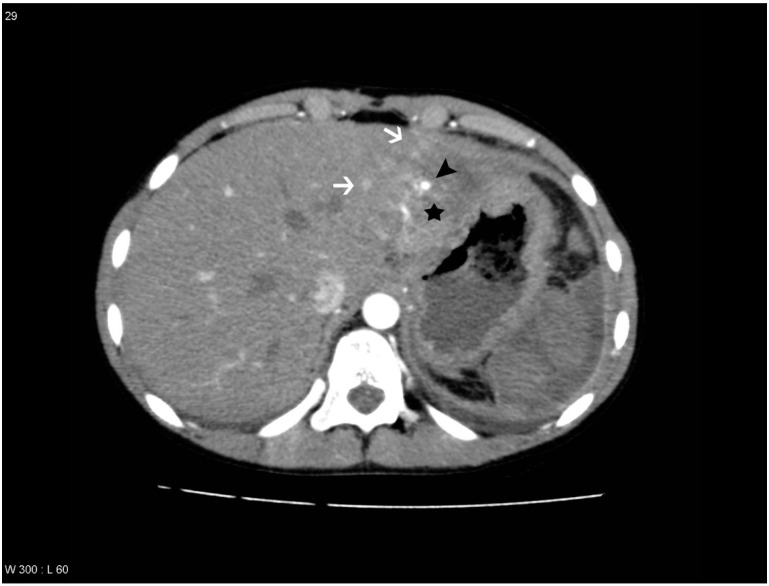
Patient with a pseudoaneurysm within the liver lesion in segment 2, which was confirmed by digital subtraction angiography (DSA) and treated with embolization. Areas with transient hepatic attenuation difference (THAD) surround the lesion. The liver lesion is marked with a black star, the areas with THAD are marked with white arrows and the pseudoaneurysm is marked with a black arrowhead.

PS/AF was defined on the arterial phase as a hyperdense focal lesion in relation to the liver lesion, with the same contrast enhancement as an adjacent contrast-enhanced hepatic artery, often surrounded by a low-attenuating rim, and on the venous phase being slightly hyperdense to isodense [2,8,9,13] (Figure 1). Gold standard for PS/AF was a previous evaluation of the follow-up CT scan done by a senior radiologist specialized in abdominal radiology with more than 20 years of experience.

**Figure 2:**
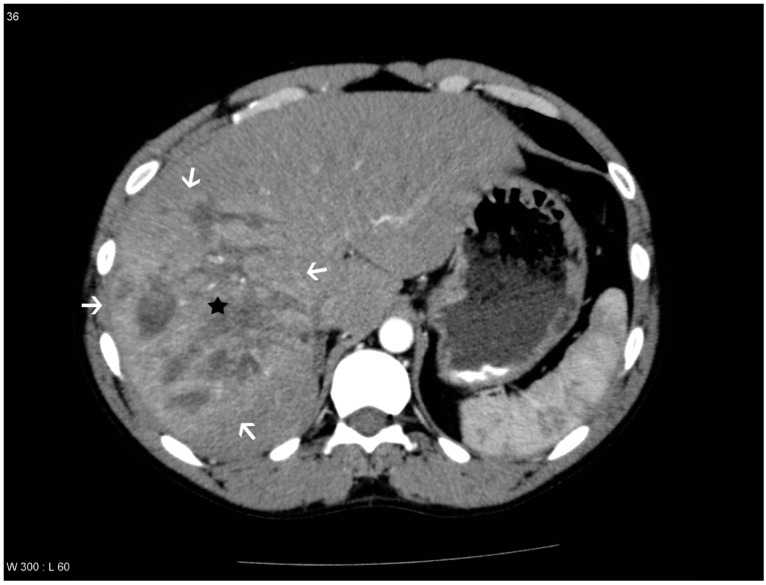
Patient with a liver lesion in segment 4a, 7 and 8. THAD is seen in the periphery of the lesion. PS/AF was not suspected and DSA was not performed. The liver lesion is marked with a black star and the areas with THAD are marked with white arrows.

**Figure 3:**
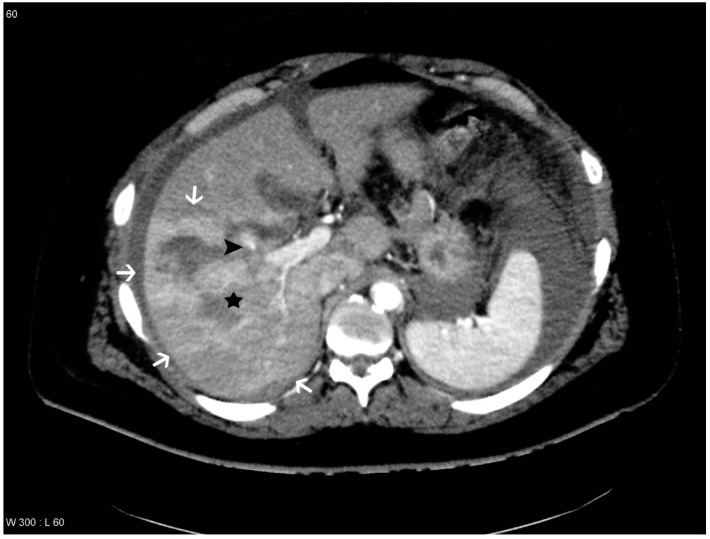
Patient with liver lesion in segment 5, 6, 7 and 8. THAD is seen in the periphery of the lesion and pseudoaneurysm and arteriovenous fistula (PS/AF) is not present according to the gold standard. However, one observer suspected a pseudoaneurysm, which is marked with a black arrowhead. The liver lesion is marked with a black star and the areas with THAD are marked with white arrows.

**Figure 4:**
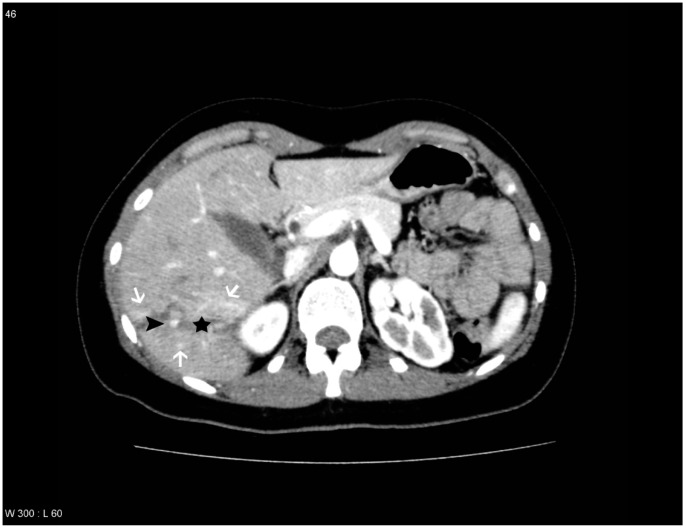
Patient with liver lesion in segment 5, 6, 7 and 8. PS/AF is seen within the lesion and THAD around the lesion. One observer missed the PS/AF. The liver lesion is marked with a black star, the areas with THAD are marked with white arrows and the pseudoaneurysm is marked with a black arrowhead.

THAD was defined as an increased arterial enhancement in relation to the liver lesion without resemblance of PS/AF. THAD after liver trauma has previously been described as a localized arterial enhancement with a polymorphous appearance [16,18]. The senior radiologist did not comment on THAD in the initial reading, primarily because THAD is regarded as a benign phenomenon. Therefore, presence of THAD was defined by a consensus between at least two out of three observers (residents in radiology) (Figure 1, Figure 2, Figure 3 and Figure 4).

Moreover, HU values were measured within the areas with THAD and in PS/AF, as well as in normal liver parenchyma and in the aorta on the follow-up CT scan. As wrong timing can affect the contrast enhancement in the arterial phase and thereby reduce the HU levels, additional ratios with HU level of the aorta as reference were calculated for each patient. Finally, The American Association for the Surgery of Trauma (AAST) organ injury scale was reported along with the number of injured Coineaud liver segments for each patient by inspecting the initial CT scan for each patient [1,3,14]. The liver lesions were identified as hypodense areas on both the initial and the follow-up CT scans.

### 2.4. Statistics

Descriptive statistics were applied to the evaluations of the three observers, the measured HUs and the location and extent of the lesions. A Generalized Estimating Equation (GEE) model was applied to estimate the likelihood of agreement when evaluating for PS/AF between the observers and the gold standard. Analysis of variance (ANOVA) and Bonferroni adjusted tests for multiple comparisons were applied to compare the HU values obtained from the follow-up CT scan in arterial phase in normal liver parenchyma, in areas with THAD, in PS/AF and in the abdominal aorta. The same statistical analyses were applied to the calculated ratios. Odds ratio and chi-square statistics were computed to investigate the association between THAD and PS/AF, and between THAD and DSA. Fischer Inter-observer variability was investigated using Fleiss’ kappa. Statistical significance level was set at 0.05. Statistical analyses were calculated using Microsoft Excel (Redmond, WA, USA), SAS (SAS Institute Inc., Cary, NC, USA) and SPSS (*SPSS* Inc., Chicago, IL, USA).

## 3. Results

Eleven patients (14%) had PS/AF according to the gold standard and of these, one was a pediatric patient. Seven patients (9%) with diagnosed PS/AF on the follow-up CT were referred to DSA and in six of these patients (8%) PS/AF was confirmed and the patients underwent intervention. The results of the ratings given by the observers when compared to the gold standard are shown in Table 1. Forty-two patients showed THAD on the follow-up CT scan and of these, 11 were pediatric patients. When applying the GEE model on data it was shown that the likelihood of agreement between the observers and the gold standard when evaluating for PS/AF was 89% when THAD was present, and 98% when THAD was absent (*p =* 0.04). The inter-observer variability for ratings by the observers showed moderate agreement for both THAD and PS/AF with kappa values of 0.41 and 0.58, respectively).

Mean HU and ratios along with standard deviations (SD) are shown in Table 2. Significant differences were present for HU measurements (*p <* 0.0001) and calculated ratios (*p <* 0.0001). Bonferroni adjusted tests for multiple comparisons for HU measurements and calculated ratios showed likewise significant differences for all comparisons (*p <* 0.05 and *p <* 0.01).

**Table 1:** Summed results for all three observers compared with the gold standard and shown for all patients, for patients with THAD and for patients without THAD. Mean sensitivity and specificity for the three observers are given for each comparison.

Patients	True Positive	False Negative	True Negative	False Positive	Sensitivity	Specificity
All patients (*n =* 234)	23	10	194	7	69.7%	96.5%
Patients with THAD (*n =* 126)	17	9	97	5	65.5%	95.0%
Patients without THAD (*n =* 108)	6	1	97	2	85.7%	98.0%

**Table 2:** Mean values and calculated ratios along with SD.

HU measured	HU (SD)	Ratio calculated	Ratio (SD)
Normal liver parenchyma	75.3 (18.8)	Normal liver parenchyma/aorta	0.27 (0.10)
THAD	100.3 (20.9)	THAD/aorta	0.33 (0.12)
PS/AF	170.3 (64.4)	PS/AF/aorta	0.51 (0.20)
Aorta	299.3 (79.1)	-	-

The location and extent of liver trauma are given in Table 3 and Table 4, respectively. The relation between AAST score and PS/AF and THAD are given in Table 4. Odds ratio and chi-square test was calculated to evaluate whether patients with THAD were susceptible to PS/AF. Odds ratio was 2.6 (CI: 0.6 to 10.5, *p =* 0.18), thus the association was not significant. To determine the relationship between performed DSA and THAD among patients with suspected PS/AF, an odds ratio and chi-square test was performed. Odds ratio was 0.14 (CI: 0.01 to 3.65, *p =* 0.12), *i.e*., not significant.

**Table 3:** Distribution of liver lesions for all patients defined by the Coineaud liver segments.

Liver Segments Involved	1	2	3	4a	4b	5	6	7	8
Injured segments (% of all injured segments) (*n =* 172)	3 (2%)	8 (4%)	11 (6%)	25 (15%)	25 (15%)	26 (15%)	24 (14%)	22 (13%)	28 (16%)

**Table 4:** Distribution of American Association for the Surgery of Trauma (AAST) organ injury scale for all patients, for patients with THAD, for patients with PS/AF and for patients with PS/AF and THAD.

Organ Injury Scale (AAST)	All Patients (% of Total)	Patients with THAD (% of all Patients of Each AAST Score)	Patients with PS/AF (% of All Patients of Each AAST Score)	Patients with PS/AF and THAD (% of All Patients of Each AAST Score)
1	0 (0%)	0 (0%)	0 (0%)	0 (0%)
2	8 (10%)	4 (50%)	0 (0%)	0 (0%)
3	39 (50%)	16 (41%)	5 (13%)	3 (8%)
4	22 (28%)	16 (72%)	4 (18%)	3 (14%)
5	9 (12%)	6 (67%)	2 (22%)	2 (22%)
Total	78 (100%)	-	-	-

## 4. Discussion

The observers diagnosed PS/AF on the follow-up CT scan with an averaged sensitivity of 69.7% and specificity of 96.5% when compared to the gold standard. However, different sensitivities and specificities were found if THAD was included as a variable. If THAD was absent on the follow-up CT scan the averaged sensitivity and specificity were 85.7% and 98.0%, while the averaged sensitivity and specificity decreased to 65.5% and 95.0% if THAD was present (Table 1). This indicates that THAD on the follow-up CT scan can result in both false positive and false negative observations when looking for PS/AF after blunt liver trauma (Figure 3 and Figure 4). Furthermore, it was shown that THAD in this study had a significant influence on the diagnosis of PS/AF for the three residents, as the likelihood of agreement for evaluation of PS/AF between the observers and the gold standard was 89% when THAD was present, and 98% when THAD was absent (*p =* 0.04).

PS/AF can be recognized and distinguished from THAD by the location, the appearance, and the HU level. PS/AFs seen in 14% of the patients were all oval or round except one, which was linear, and all had focal enhancement with mean HU levels of 170. All PS/AFs were found within the liver lesions (Figure 1 and Figure 4). PS and AF were indistinguishable as stated previously by others [9]. Areas with THAD seen in 54% of the patients had hazy borders, were all found in the periphery of the lesion and with mean HU levels of 100 (Figure 1, Figure 2, Figure 3 and Figure 4).

HU was measured in areas with THAD, in PS/AF, in normal liver parenchyma and in the abdominal aorta (Table 2). Willmann *et al*. [19] found a mean HU attenuation of 155 in active bleeding after blunt hepatic trauma, which is comparable to our HU measurements of PS/AF. The HU levels were significantly different in the four locations with successively increasing HU levels from normal liver parenchyma, areas with THAD, PS/AF to the abdominal aorta (*p <* 0.0001). Moreover, calculated ratios, compensating for differences in arterial enhancement, also showed significant differences (*p <* 0.0001). A threshold for HU in PS/AF should ensure a high sensitivity, and include the lower 95% confidence interval (CI), which in this study corresponds to HU of 96. Only eleven patients were used for calculating mean HU of PS/AF, which affects CI and threshold. More data is needed before a threshold can be determined, as the mean HU of THAD of 100 is overlapping the threshold for PS/AF.

We found a higher prevalence for PS/AF and THAD with higher AAST score (Table 4). This is in concordance with other studies reporting higher prevalence for PS/AF among patients with higher AAST score [7,10,20,21]. THAD is a localized reaction in the periphery of the liver lesion due to parenchymal inflammation and portal inflow obstruction, which probably are correlated to higher AAST scores as indicated in this study [18]. Of the patients with PS/AF, 72% had areas with THAD while only 45% of the patients without PS/AF showed THAD. Even though, the difference between the two groups was not significant (*p =* 0.18), probably due to the relatively small study population, the study indicates a correlation between THAD and PS/AF.

The right hepatic lobe is a frequent site of injury, because of its size and the fixation of the posterior, superior segments close to the spine and ribs. Traumatic lesions to the left hepatic lobe are rare and usually associated with direct trauma to the abdomen. Lesions to the caudal liver segments are extremely rare and often associated with large parenchymal lesions [22,23]. We obtained similar results, with most lesions being in the right hepatic lobe (58%), followed by the left lobe (40%) and only very few lesions in the caudal liver segment (2%) (Table 3).

An experienced senior radiologist, who served as gold standard, diagnosed PS/AF on the follow-up CT scans in 14% of the patients with blunt hepatic trauma. Previous studies have reported PS/AF and liver-related failures after non-surgical management of blunt hepatic trauma in 1.2% to 4.1% of the patients [7,10,21]. Cox *et al*. [20] reported that only 0.5% underwent intervention due to PS/AF after blunt hepatic trauma. In our study seven patients were referred to DSA and of these, six patients (7.7%) had PS/AF confirmed and underwent intervention. Thus, one patient was suspected for PS/AF, but showed no signs of DVC on the DSA, which is not uncommon due to, e.g., vaso-spasm or vessel truncation [8]. Four patients did not undergo DSA even though PS/AF was suspected on the CT. They all had normal hemoglobin values, normal blood pressure and were clinically stable without abdominal pain. Three of the four patients were evaluated with an additional CT scan one month later showing no DVC. The last patient with suspected PS/AF was not further evaluated, as the patient recovered rapidly and had no abdominal complaints. Furthermore, the appearance of THAD could not be used to distinguish which patients needed DSA (*p =* 0.12).

The higher prevalence of PS/AF in this study compared with the literature could be a consequence of improved imaging techniques, using modern 64-slice-helical CT scanners and reconstruction algorithms. Moreover, as Copenhagen University Hospital, Rigshospitalet is a level 1 trauma center; the patient population was highly selected with higher AAST score and therefore, at higher risk for developing PS/AF compared to previous studied patient populations [7,10,20,21]. In this study, 54% of the study population showed THAD. Colagrande *et al*. [24] identified 13% with THAD among 988 patients. However, the study population included both patients with and without liver lesions.

Some limitations of this study have to be mentioned. The initial CT scans were performed at different hospitals using different protocols, which may influence the retrospective assessment of AAST score and involved liver segments. DSA was not performed in all patients and therefore, the gold standard was the initial evaluation of the follow-up CT scan done by a senior radiologist specialized in abdominal radiology. This could have resulted in both false positive and false negative evaluations and can explain a part of the discrepancy in prevalence of PS/AF found in this study compared with previous studies [10,21]. The retrospective study design did not allow for parameter changes and additional reconstructions on the follow-up CT scans. An obvious reconstruction technique to be investigated is maximum intensity projection (MIP) as this technique provides increased visualization of vessels and has been shown to improve the recognition of intracranial aneurysms [25,26,27]. MIP reconstructions will be addressed in future prospective studies concerning detection of PS/AF after liver trauma on follow-up CT scan. The long-term outcome for patients with blunt liver trauma and presence of THAD on follow-up CT is not investigated in this study but will be addressed in future studies.

## 5. Conclusions

THAD is a common phenomenon on the follow-up CT scan after liver trauma and can reduce the diagnostic accuracy for detection of PS/AF for the less trained observer, e.g., the resident in radiology. Furthermore, the study indicates that THAD is correlated to higher AAST score and the presence of PS/AF. Location, appearance and HU level may be useful diagnostic parameters to make the distinction between PS/AF and THAD.
